# Paraoxonase 1 activity in the sperm-rich portion of boar ejaculates is positively associated with sperm quality

**DOI:** 10.1590/1984-3143-AR2022-0039

**Published:** 2022-09-12

**Authors:** Matheus Schardong Lucca, Karina Lemos Goularte, Monique Tomazele Rovani, Augusto Schneider, Bernardo Garziera Gasperin, Thomaz Lucia, Carlos Augusto Rigon Rossi

**Affiliations:** 1 Androlab, Faculdade de Medicina Veterinária, Universidade Federal de Santa Maria, Santa Maria, RS, Brasil; 2 ReproPEL, Faculdade de Veterinária, Faculdade de Nutrição, Universidade Federal de Pelotas, Pelotas, RS, Brasil; 3 Departamento de Medicina Animal, Faculdade de Veterinária, Universidade Federal do Rio Grande do Sul, Porto Alegre, RS, Brasil; 4 Departamento de Nutrição, Faculdade de Nutrição, Universidade Federal de Pelotas, Pelotas, RS, Brasil

**Keywords:** PON1, spermatozoa kinetics, membrane integrity, DNA integrity

## Abstract

Associations of the activity of the paraoxonase 1 (PON1) enzyme with boar sperm quality still needs to be characterized, since boar ejaculates present distinct portions with differences in sperm concentration and quality. This study evaluated PON1 activity in the serum, in the distinct portions of boar ejaculates and estimated correlations with sperm quality parameters. Ejaculates and blood samples were collected from six boars for three weeks (two per week per boar; *n* = 36). Serum and post-spermatic portion PON1 activities were positively correlated (*P* = 0.01) but were both uncorrelated with the PON1 activity in the sperm-rich portion and in the whole ejaculate (*P* > 0.05). Differences in PON1 activity among boars were only observed in the sperm-rich portion of the ejaculate (*P* < 0.05). The PON1 activity in the serum and in the post-spermatic portion was generally negatively correlated with parameters of spermatozoa kinetics (*P* < 0.05). In the sperm-rich portion, PON1 activity was positively correlated with sperm concentration (*P* < 0.0001), curvilinear distance and velocity (both *P* < 0.05) and DNA integrity (*P* < 0.05), but negatively correlated with straightness and linearity (*P* < 0.05). Thus, boar ejaculates with increased PON1 activity in the sperm-rich portion may present increased concentration and spermatozoa with acceptable curvilinear velocity and distance and DNA integrity, which suggests that PON1 activity may be a biomarker for potential fertility.

## Introduction

In the artificial insemination (AI) programs currently employed in commercial swine farms, most of the services tend to be concentrated on a reduced number of boars (Waberski et al., 2019). Ideally, those would be the boars having the greatest fertility. Nonetheless, not only there is great variation among individuals (Broekhuijse et al., 2012a; Ferreira et al., 2015), but also the available methods to classify boars according to their sperm quality and potential fertility are not precisely correlated with their field performance (Dyck et al., 2011; Broekhuijse et al., 2012b). Therefore, accurate methods to screen highly fertile boars are still required.

Cooled boar sperm can be kept at 15-17 °C for up to 3 d, with short-term extenders, and up to 7 d, with long-term extenders (Knox, 2016). During such periods, boar spermatozoa may be exposed to oxidative stress-induced injuries, attributed to the production of reactive oxygen species (ROS), which may impair their fertilizing capacity. Although ROS are related to a variety of physiological roles, such as sperm capacitation control (Peña et al., 2004), their production in excess may lead to peroxidative damages on spermatozoa membranes and DNA (Guthrie and Welch, 2012). Even with inclusion of antioxidants in the extenders, boar spermatozoa are still susceptible to such injuries due to the content of polyunsaturated fatty acids in their phospholipid bilayer membrane, which is greater compared to other domestic species (Cerolini et al., 2000; Awda et al., 2009), and also because they have limited intracellular antioxidant mechanisms (Brezezińska-Slebodzińska et al., 1995).

The paraoxonase 1 (PON1) is an enzyme mainly synthesized by the liver and secreted into the blood (Ferré et al., 2002), which has a wide range of substrates (Aşkar and Büyükleblebici, 2012) and presents antioxidant properties (Précourt et al., 2011). In the serum, PON1 is associated with high-density lipoprotein (HDL-C) (Mackness et al., 1998). The PON1 has been identified in the seminal plasma of men (Verit et al., 2009), roosters (Khan et al., 2012), bulls (Ferreira et al., 2018) and boars (Barranco et al., 2015a). In boars, the PON1 is expressed in the epididymis epithelium and in the secretory epithelium of the accessory sex glands (Barranco et al., 2017). Due to its associations with reproductive functions in various species, PON1 has been considered as a potential fertility biomarker. Reduced PON1 activity was observed in the seminal plasma of men presenting teratozoospermia, azoospermia, subfertility and infertility (Verit et al., 2009; Gulum et al., 2017; Ozer et al., 2019), whereas increased PON1 activity was positively associated with sperm concentration (Ozer et al., 2019). In bulls, PON1 activity in the seminal plasma presented positive correlations with a subjective indicator of sperm concentration and with several parameters of sperm quality (Ferreira et al., 2018). In boars, greater PON1 activity in the seminal plasma was positively correlated with sperm concentration (Barranco et al., 2015b) and associated with increased motility, reduced production of ROS (Barranco et al., 2015a), improved cryotolerance (Li et al., 2018) and increased farrowing rates after AI (Barranco et al., 2015b).

However, unlike other species, boar ejaculates present portions with distinct sperm concentrations (Peña et al., 2006). The portion with the greatest concentration (sperm-rich portion) also presents distinct seminal plasma protein content and better response to cryopreservation than both the other portions and the whole ejaculate (Peña et al., 2004, 2006; Corcini et al., 2012). Additionally, the sperm-rich portion presents the greatest PON1 activity than the other portions of boar ejaculates (Barranco et al., 2015b). Thus, the effects of the PON1 activity on boar sperm quality still need to be further investigated, considering the different characteristics of distinct portions of boar ejaculates. This study aimed to evaluate the PON1 activity in the serum and in different portions of the ejaculates of boars, and to investigate their association with parameters of sperm quality.

## Methods

All procedures involving animals were approved by the Ethics Committee of both the Universidade Federal de Santa Maria (Act #135) and the Universidade Federal de Pelotas (Act #36040).

### Animals, semen collection and processing

Six sexually mature crossbred boars (Landrace x Large White), aged between 18 and 30 months old, from the same genetic supplier were used. The boars were submitted to a weekly routine of sperm collection and were previously known to have acceptable parameters of sperm quality (motility and normal morphology of at least 70%). They were housed in individual pens at the Universidade Federal de Pelotas, fed 2.8 kg/d of a commercial diet (3,000 kcal, 16% crude protein and 0.6% digestible lysine) and had *ad libitum* access to water.

For three weeks, ejaculates were collected from those boars, twice a week, within 3-d intervals, totaling 36 ejaculates (six per boar), using the gloved hand technique. At the time of collection, ejaculates were placed in 500 mL plastic containers kept at 35 °C and split in two portions: first, the sperm-rich portion was placed in a pre-warmed flask (56.6 mL, in average); then, the post-spermatic portion was conditioned in another pre-warmed flask (179.1 mL, in average). A 2 mL aliquot was taken from each portion. Thereafter, both portions (were mixed to compose the whole ejaculate (231.7 mL, in average), from which an additional 2 mL aliquot was taken. Sperm concentration was determined using a Neubauer chamber. The whole ejaculate was extended in EoBOS® (Gestión Veterinaria Porcina, Madrid, Spain) producing semen doses with 3 x 10^9^ sperm cells and stored in blister (Minitube GmbH, Tiefenbach, Germany) with total volume of 80 mL. After 90 min from final dilution, the semen doses were stored in a controlled-temperature cabinet at 17 ± 1 °C for 24h. Evaluations of spermatozoa kinetics and viability were subsequently conducted considering the whole ejaculate extended.

### Blood collections

Blood samples (5 mL) were collected from the jugular vein of each boar with a 16 G needle, after each semen collection, totalizing 36 samples (six per boar). Samples were centrifuged at 1,800 x g for 5 min at 10 °C, to separate the serum, and were then stored at -20 °C, until the evaluation of PON1 activity.

### Spermatozoa kinetics analyses

These analyses were conducted using a computer-assisted semen analysis system (CASA system; Sperm Vision® 3.7; MOFA Global, Verona, WI, USA). Aliquots of 1 mL of extended boar semen were incubated at 37 °C for 10 min. A 3 µL aliquot of each sample was placed by capillarity in a 20 µm chamber (Leja®, Nieuw Vennep, The Netherlands) and analyzed in a phase contrast microscope (Axio Scope A1, Zeiss®, Oberkochen, Germany) at 200 x. A minimum of 1,000 sperm cells were analyzed per sample within six centrally automated randomized fields, each with a rate of 60 frames per second. The CASA System settings were: cell detection with minimum head size of 18 and a maximum of 120 mm. Sperm cells were classified as motile if the average orientation change (AOC) was ≥3.0 mm, and as progressively motile, if the distance straight line (DSL) was ≥4.5 mm. The coefficient of variation among field concentrations (total number of cells counted per field) was lower than 15%. The evaluated parameters were: total and progressive motility; straight (DSL) and curvilinear (DCL) distance; average distance path (DAP); straight (VSL) and curvilinear (VCL) velocity; average path velocity (VAP); amplitude of lateral head displacement (ALH); linearity (LIN); straightness (STR), beat cross frequency (BCF) and wobble (WOB).

### Spermatozoa viability and function analyses

Those analyses were conducted using an epifluorescence microscope (Olympus BX 51, América INC, São Paulo, Brazil) with a WU filter (excitation of 450-490 nm and emission of 516-617 nm). All chemicals used were from Sigma Chemical Company (St. Louis, MO, USA). The samples having 5 × 10^4^ spermatozoa in 0.05 ml were incubated at 37 °C for 10 min, and two hundred sperm cells were evaluated per slide.

Membrane integrity was evaluated using 0.46 mg mL^-1^ fluorescent carboxy fluorescein diacetate and 0.5 mg mL^-1^ propidium iodide (Harrison and Vickers, 1990). Spermatozoa with damaged membranes were stained with either red fluorescence or both green and red fluorescence, whereas intact membranes were only stained with green fluorescence.

Mitochondrial functionality was evaluated using 0.53 mM rhodamine 123 and propidium iodide (Garner et al., 1997). The cells that presented the middle piece with intense green fluorescence were considered to have intact mitochondria (functionality active), while cells without or with a low green fluorescence intensity were considered non-intact.

Lectin from *Arachis hypogaea* FITC (fluorescein isothiocyanate) conjugate (100 μg mL^-1^) was used for analyses of acrosome integrity (Kawamoto et al., 1999). Intact acrosomes remained unstained. Spermatozoa with damaged acrosomes showed green fluorescence.

The DNA integrity was assessed using 6 μg mL^-1^ acridine orange (Evenson et al. (1999). Orange fluorescence-stained spermatozoa with denatured DNA. Spermatozoa with intact DNA were stained with green fluorescence.

### Measurement of PON1 activity

Samples from serum, the sperm-rich portion, post-spermatic portion, and from the whole ejaculate were centrifuged at 10,000 x g for 10 min, to separate the cells and this supernatant were used for PON1 analyses. The PON1 arylesterase activity was determined as described by Browne et al. (2007). Briefly, samples were diluted 1:3 in a 20 mM Tris/HCl buffer solution (1 mM CaCl_2_). The absorbance was measured at 270 nm through spectrophotometry (Cirrus 80MB; FEMTO, São Paulo, Brazil) for 60 s, using 3,3 μl of the diluted samples in 500 μl of the buffer (containing 4 mM phenylacetate). The activity, expressed in U/mL, was determined by the formula: Δ absorbance*115*3.

### Statistical analysis

After checking the responses of interest for normality with the Shapiro-Wilk test, transformations were applied, whenever necessary. Sperm concentration was normalized through transformation to the logarithmic scale, but no transformation normalized the distribution of the activity of paraoxonase 1 in distinct portions of boar ejaculates.

The PON1 activity in serum and in distinct portions of the ejaculates, parameters of spermatozoa kinetics, and viability were compared among boars through analyses of variance, including the effect of the date of semen collection as a covariate (to adjust the repeated effect of various collections per boar). Comparisons of means were done by the Tukey test. Comparisons involving PON1 activity in distinct portions of boar ejaculates were conducted with the Kruskal-Wallis analyses of variance for non-parametric data, but reported in the original scale, for interpretation.

Correlations among PON1 activity and sperm quality parameters were estimated by Pearson’s coefficients, when both variables presented normality, or by Spearman’s rank coefficients for non-parametric data, for comparisons involving the PON1 activity in distinct portions of boar ejaculates. All statistical analyses were conducted with Statistix® (2013).

## Results

Across boars, there were no differences (*P* < 0.05) on the PON1 activity in the blood serum, in distinct portions of ejaculates and whole ejaculate (Table 1). The PON1 activity differed among boars only within the sperm-rich portion, in which it was greater for Boars 2 and 3 than for Boars 4, 5 and 6 (*P* < 0.05).

**Table 1:** Activity of paraoxonase 1 in the blood serum, in distinct portions of ejaculates and in the whole ejaculates of boars (*n* = 36; 6 boars x 6 collections).

**Boars**	**Paraoxonase 1 activity (UI mL^-1^)**			
	**Blood Serum**	**Post-spermatic portion**	**Sperm-rich portion**	**Whole ejaculate**
1	6.0 ± 1.4	0.14 ± 0.40	3.1 ± 0.4^AB^	1.9 ± 0.5
2	8.8 ± 1.0	1.36 ± 0.31	3.3 ± 0.3^A^	2.1 ± 0.3
3	9.1 ± 0.9	1.09 ± 0.28	3.3 ± 0.3^A^	2.2 ± 0.4
4	9.7 ± 0.9	0.63 ± 0.29	1.5 ± 0.3^BC^	1.3 ± 0.4
5	9.7 ± 0.9	0.74 ± 0.28	1.6 ± 0.3^BC^	1.2 ± 0.3
6	10.2 ± 1.1	0.86 ± 0.29	1.0 ± 0.3^C^	1.2 ± 0.3
Total	9.2 ± 2.4	0.85 ± 0.72	2.2 ± 1.2	1.6 ± 0.9

^A,B,C^Means ± standard errors of the mean with distinct superscripts differ *P* < 0.05.

As shown in Table 2, sperm concentration was greater for Boars 2 and 3 than for Boars 5 and 6 (*P* < 0.05), whereas Boars 1 and 4 presented intermediate concentrations that did not differ from those of any other boars (*P* > 0.05). The only parameter of spermatozoa kinetics that differed among boars was BCF, which was greater for Boar 1 (*P* > 0.05) than for Boars 3, 5 and 6 (Table 2). No parameter of spermatozoa viability and function differed across boars (*P* > 0.05, Table 3).

**Table 2:** Parameters of sperm concentration and kinetics for different boars in semen doses after 24 h storage (*n* = 36; 6 boars x 6 collections).

**Parameter**	**Boars**					
	**1**	**2**	**3**	**4**	**5**	**6**
Sperm concentration/mL (x 10^9^)	3.6 ± 1.2^AB^	5.8 ± 0.9^A^	5,4 ± 0.9^A^	3.1 ± 0.9^AB^	2.0 ± 0.9^B^	1.5 ± 0.9^B^
Total motility (%)	84.2 ± 6.6	80.6 ± 5.1	74.2 ± 4.6	80.4 ± 4.3	78.5 ± 4.5	73.9 ± 4.6
Progressive motility (%)	71.3 ± 8.1	66.9 ± 6.3	54.9 ± 5.7	62.8 ± 5.5	63.5 ± 5.6	52.1 ± 5.8
Distance average path (µm)	20.1 ± 1.6	23.1 ± 1.2	20.3 ± 1.1	19.8 ± 1.1	22.9 ± 1.1	18.8 ± 1.1
Distance in a curved line (µm)	38.3 ± 3.2	44.7 ± 2.4	40.5 ± 2.2	39.5 ± 2.2	43.7 ± 2.2	35.0 ± 2.2
Distance in a straight line (µm)	15.4 ± 1.0	15.6 ± 0.8	13.9 ± 0.7	14.0 ± 0.7	15.9 ± 0.7	14.2 ± 0.7
Average path velocity (µm/s)	42.6 ± 3.9	51.3 ± 3.0	44.3 ± 2.7	42.7 ± 2,6	49.9 ± 2,6	40.4 ± 3,1
Velocity in a curved line (µm/s)	81.1 ± 7.1	98.6 ± 5.4	87.6 ± 5.9	84.6 ± 5.7	94.8 ± 5.6	74.9 ± 5,8
Velocity in a straight line (µm/s)	32.7 ± 2.3	34.5 ± 1.7	30.1 ± 1.6	30.2 ± 1.4	34.7 ± 1.5	30.6 ± 1.6
Straightness (%)	75.9 ± 2.6	67.2 ± 2.0	68.4 ± 1.8	70.6 ± 1.9	69.8 ± 1.9	75.4 ± 1.9
Linearity (%)	39.9 ± 1.8	34.8 ± 1.4	34.2 ± 1.3	35,5 ± 1.3	36.5 ± 1.3	40.2 ± 1.3
Amplitude of lateral head displacement (%)	2.1 ± 0.3	3.3 ± 0.2	2.9 ± 0.2	2.7 ± 0.2	2.9 ± 0.2	2.4 ± 0.2
Beat cross frequency (%)	37.0 ± 1.1^A^	33.2 ± 0.9^AB^	31.8 ± 0.8^B^	32.3 ± 0.8 ^B^	33.5 ± 0.8^AB^	31.9 ± 0.8^B^
Wobble (%)	0.52 ± 0.02	0.51 ± 0.01	0.50 ± 0.01	0.50 ± 0.01	0.52 ± 0.01	0.53 ± 0.01

^AB^Means ± standard errors of the mean with distinct superscripts differ *P* < 0.05.

**Table 3:** Parameters of spermatozoa viability and function for different boars in semen doses after 24 h storage (*n* = 36; 6 boars x 6 collections).

**Parameter**	**Boars**					
	**1**	**2**	**3**	**4**	**5**	**6**
DNA integrity (%)	99.8 ± 6.2	95.4 ± 4.8	94.9 ± 4.4	90.7 ± 4.4	98.0 ± 4.2	94.1 ± 4.3
Acrosome integrity (%)	95.6 ± 1.0	86.4 ± 8.6	92.7 ± 7.8	84.6 ± 8.0	80.2 ± 7.9	84.2 ± 7.8
Membrane integrity (%)	89.1 ± 4.7	75.9 ± 3.6	90.7 ± 3.3	82.0 ± 3.2	81.1 ± 3.3	83.4 ± 3.0
Mitochondrial functionality (%)	89.7 ± 8.2	63.9 ± 6.3	86.4 ± 5.7	80.3 ± 5.6	71.2 ± 5.7	73.9 ± 5.8

^AB^Means ± standard errors of the mean with distinct superscripts differ *P* < 0.05.

Although the PON1 activity in the serum was positively correlated with the PON1 activity in the post-spermatic portion (r = 0.45, *P* = 0.01), it was not correlated (*P* > 0.05) with the activity in both the sperm-rich portion (r = -0.30) and in the whole ejaculate (r = 0.03). Additionally, the PON1 activity in the sperm-rich portion and in the whole ejaculate were positively correlated (r = 0.49, *P* = 0.005).

**Figure 1:**
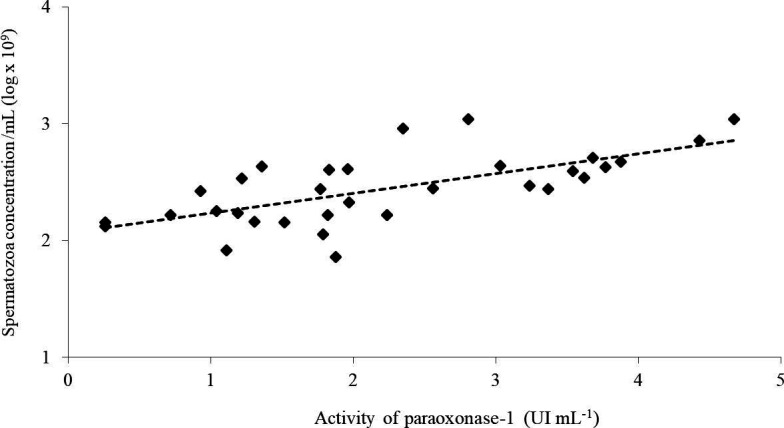
Correlation (Spearman rank r = 0.67, P < 0.0001) between activity of paraoxonase type 1 and spermatozoa concentration (x log) in the sperm-rich portion of boar ejaculates (n = 36; 6 boars x 6 collections).

The PON1 activity in the sperm-rich portion of the ejaculates presented a positive correlation (r = 0.69, *P* < 0.0001) with sperm concentration (Figure 1). However, spermatozoa concentration was not correlated (*P* > 0.05) with the PON1 activity in the serum (r = -0.32), in the post-spermatic portion (r = -0.11) and in the whole ejaculate (r = 0.26).

The serum PON1 activity was negatively correlated (*P* < 0.05) with progressive sperm motility, DAP, DSL, VAP and VSL (Table 4) and the PON1 activity in the post-spermatic portion was negatively correlated with progressive motility, DSL and VSL (*P* < 0.05). On the other hand, the PON1 activity in the sperm-rich portion of ejaculates was positively correlated with DCL and VCL (*P* < 0.05), but negatively correlated with STR and LIN (*P* < 0.05). No spermatozoa kinetics parameters were correlated with PON1 activity in the whole ejaculate (*P* > 0.05).

**Table 4:** Correlation coefficients (r) among the activity of paraoxonase type 1 in the serum, in distinct portions of ejaculates and in the whole ejaculates of boars with parameters of spermatozoa kinetics (*n* = 36: 6 boars x 6 collections).

**Parameter**	Mean ± SD^*^	**Paraoxonase 1 activity**			
		**Blood Serum**	**Post-spermatic portion^†^**	**Sperm-rich portion**	**Whole ejaculate**
Total motility (%)	78.6 ± 10.6	-0.31 (*P* = 0.08)	-0.34 (*P* = 0.06)	0.11 (*P* = 0.53)	-0.14 (*P* = 0.43)
Progressive motility (%)	62.0 ± 14.0	-0.51 (*P* = 0.03)	-0.35 (*P* = 0.05)	0.11 (*P* = 0.59)	-0.26 (*P* = 0.14)
Distance average path (µm)	20.8 ± 3.0	-0.36 (*P* = 0.04)	-0.18 (*P* = 0.32)	0.28 (*P* = 0.11)	-0.10 (*P* = 0.58)
Distance in a curved line (µm)	40.3 ± 5.9	-0.24 (*P* = 0.18)	-0.11 (*P* = 0.55)	0.35 (*P* = 0.05)	-0.01 (*P* = 0.96)
Distance in a straight line (µm)	14.7 ± 1.7	-0.35 (*P* = 0.04)	-0.36 (*P* = 0.04)	0.12 (*P* = 0.49)	-0.27 (*P* = 0.13)
Average path velocity (µm/s)	45.2 ± 7.4	-0.39 (*P* = 0.03)	-0.16 (*P* = 0.36)	0.29 (*P* = 0.12)	-0.08 (*P* = 0.65)
Velocity in a curved line (µm/s)	87.0 ± 13.4	-0.29 (*P* = 0.10)	-0.10 (*P* = 0.59)	0.37 (*P* = 0.04)	-0.01 (*P* = 0.99)
Velocity in a straight line (µm/s)	32.2 ± 4.0	-0.42 (*P* = 0.02)	-0.34 (*P* = 0.06)	0.16 (*P* = 0.37)	-0.25 (*P* = 0.17)
Straightness (%)	71.0 ± 0.05	0.16 (*P* = 0.39)	-0.04 (*P* = 0.81)	-0.36 (*P* = 0.04)	-0.21 (*P* = 0.26)
Linearity (%)	37.0 ± 0.03	-0.12 (*P* = 0.56)	-0.28 (*P* = 0.12)	-0.37 (*P* = 0.04)	-0.32 (*P* = 0.08)
Amplitude of lateral head displacement (%)	2.7 ± 0.6	-0.24 (*P* = 0.19)	-0.02 (*P* = 0.91)	0.32 (*P* = 0.08)	-0.06 (*P* = 0.72)
Beat cross frequency (%)	32.9 ± 2.3	-0.34 (*P* = 0.06)	-0.11 (*P* = 0.56)	0.17 (*P* = 0.36)	-0.12 (*P* = 0.50)
Wobble (%)	0.5 ± 0.03	-0.29 (*P* = 0.11)	-0.29 (*P* = 0.11)	-0.17 (*P* = 0.35)	-0.21 (*P* = 0.24)

* Standard deviation; †Spearman rank correlation coefficients for non-parametric data.

Spermatozoa DNA integrity was the only parameter of sperm viability and function correlated with the PON1 activity (Table 5). Although it was negatively correlated with serum PON1 activity, spermatozoa DNA integrity was positively correlated with PON1 activity in the sperm-rich portion of the ejaculate (both *P* < 0.05).

**Table 5:** Spearman’s rank correlation coefficients (r) for non-parametric data among the activity of paraoxonase 1 in the serum, in distinct portions of ejaculates and in the whole ejaculates of boars with parameters of spermatozoa viability (*n* = 36: 6 boars x 6 collections).

**Parameter**	**Mean ± SD** *	**Paraoxonase 1 activity**			
		**Blood Serum**	**Post-spermatic portion**	**Sperm-rich portion**	**Whole ejaculate**
DNA integrity (%)	95.4 ± 10.2	-0.37 (*P* = 0.04)	-0.19 (*P* = 0.29)	0.38 (*P* = 0.03)	0.06 (*P* = 0.76)
Acrosome integrity (%)	87.5 ± 18.7	-0.14 (*P* = 0.43)	-0.12 (*P* = 0.50)	0.18 (*P* = 0.32)	0.05 (*P* = 0.19)
Membrane integrity (%)	89.9 ± 10.6	-0.13 (*P* = 0.49)	-0.14 (*P* = 0.43)	0.15 (*P* = 0.39)	0.25 (*P* = 0.16)
Mitochondrial functionality (%)	77.7 ± 15.7	-0.07 (*P* = 0.71)	-0.20 (*P* = 0.27)	0.19 (*P* = 0.28)	0.24 (*P* = 0.18)

## Discussion

To our knowledge, this is the first study to investigate PON1 activity in the serum and in distinct portions of boar ejaculates altogether, evaluating correlations with sperm quality. This association was expected, because PON1 is mainly synthesized in the liver. Although PON1 activity in the serum was positively correlated with the PON1 activity in the post-spermatic portion, no significant correlation was observed with the sperm-rich portion and the whole ejaculate. This may be explained by the fact that PON1 is also expressed in the epididymis and accessory sex glands, and because PON1 binds to HDL, which is more abundant in the sperm-rich portion (Barranco et al., 2017). Thus, PON1 activity may vary according to the local synthesis and concentration of other compounds, such as HDL.

The observed association with sperm concentration may be due to the concomitant correlation between PON1 activity and the HDL-C concentration, which is abundant in the epididymal fluid and in the sperm-rich portion (Barranco et al., 2017), since cholesterol is a substantial component of the spermatozoa membrane (Barranco et al., 2015a, b). The HDL-C/PON1 combination is capable of hydrolyzing long-chain phospholipids (Aşkar and Büyükleblebici, 2012), which could protect spermatozoa against oxidative damages, as reported for men sperm (Verit et al., 2009). Although PON1 is mainly synthesized in the liver (Aşkar and Büyükleblebici, 2012), it is expressed in the reproductive tract of boars (Barranco et al., 2017). As PON1 is also synthesized in epididymis and accessory sex glands and secreted in the seminal plasma, its activity is probably mostly aimed to ejaculated spermatozoa. Nevertheless, the observed positive correlation with sperm concentration suggests some effect of PON1 at testicular level, which might be due to its antioxidant properties, resulting in declined intratesticular production of ROS (Verit et al., 2009). Such hypothesis still requires further investigation.

Our results suggest that spermatozoa with DNA integrity would be more likely to be present in ejaculates with increased PON1 activity in the sperm-rich portion, but also when serum PON1 activity is reduced. Although such correlations might be contradictory, they probably reflect the distinct PON1 activities in the serum and in the sperm-rich portion of the ejaculate. As reported for men sperm, higher levels of sperm DNA fragmentation are associated with a polymorphism in the PON1 gene (Ji et al., 2012) and the addition of purified PON1 to human sperm incubated in capacitation medium inhibited hyperactivation and decreased acrosome reaction (Efrat et al., 2019). As oxidative stress is one of the leading causes of spermatozoa DNA fragmentation (Aitken and De Iuliis, 2010; González-Marín et al., 2012), the positive correlation between PON1 activity in the sperm-rich portion and DNA integrity may be due to the protective role of PON1 against oxidative injuries induced by excessive production of ROS. Parameters of boar sperm viability and function, which include not only DNA fragmentation, but also membrane and acrosome integrity and mitochondrial functionality, are influenced by the protein content in the ejaculates, especially that of the sperm-rich portion (Peña et al., 2006; Corcini et al., 2012), which is associated with spermatozoa cryotolerance (Siqueira et al., 2011). However, in the present study, parameters of spermatozoa viability and function did not differ among boars and DNA integrity was the only one of such parameters correlated with PON1 activity.

Our findings indicate that, among all tested sources, the PON1 activity in the sperm-rich portion of ejaculates may be a candidate biomarker for boar fertility, as indicated elsewhere (Barranco et al., 2015b; Ferreira et al., 2018), since ejaculates with increased PON1 activity in the sperm-rich portion would be more likely to have increased sperm concentration and greater frequency of spermatozoa with DNA integrity. In the present study, the two boars presenting the greatest PON1 activity in the sperm-rich portion of their ejaculates (Boars 2 and 3) were also those presenting the greatest sperm concentration, reflecting the fact that the sperm-rich portion presents greater sperm concentration (Peña et al., 2004). Even though previously reported associations of PON1 activity with boar sperm quality showed no correlation with sperm concentration (Barranco et al., 2015b), correlation of PON1 activity with men sperm concentration was reported (Verit et al., 2009). However, in the present study, the PON1 activity in the serum, post-spermatic portion and whole ejaculate did not differ across boars and were not correlated with sperm concentration.

Despite of the lack of differences in spermatozoa kinetics across boars, in the present study, PON1 activity in distinct sources was correlated with several parameters of spermatozoa kinetics. Serum and the others distinct portions of boar ejaculates PON1 activities were negatively correlated with progressive motility and with straight velocity and distance parameters. Nevertheless, although they were positively correlated among themselves, serum and post-spermatic portion PON1 activities were both uncorrelated with the PON1 activity in the sperm-rich portion of the ejaculates. Paradoxically, in the present study, PON1 activity in the sperm-rich portion of the ejaculate was positively correlated with VCL and DCL (parameters of curvilinear movement) but negatively correlated with STR and LIN. Those might be considered negative effects, since LIN and STR reflect the spermatozoa ability to move straight and penetrate through the cervical mucus, and have been described as positively correlated with pregnancy rates (Centola, 1996; Broekhuijse et al., 2012b). On the other hand, positive correlations of VCL with boar sperm quality have been reported (Verstegen et al., 2002; Broekhuijse et al., 2011), and associated with positive effects on subsequent fertility *in vivo* (Barranco et al., 2015b; Jung et al., 2015). Additionally, Barranco et al. (2015b) reported positive correlations of PON1 activity with total and progressive motility, but not with other kinetics parameters.

Thus, those findings indicate that PON1 activity is associated with boar spermatozoa kinetics, but its effects on the quality of such movements may be highly influenced by individual variation, even among boars with similar *in vitro* sperm quality, such as those included in the present study. That could also explain the differences observed across boars in BCF, a parameter proportional to the beating frequency of the spermatozoa tail (Su et al., 2012), which presented no correlation with PON1 activity in any of the tested sources. Such individual variation may reflect the fact that PON1 activity is influenced by genetic polymorphisms, environmental and dietary factors, as recently reviewed by Ponce-Ruiz et al. (2020). Also, it was recently demonstrated that phenotypic distribution can affect seminal plasma PON1 activity in men (Ozer et al., 2019).

## Conclusion

The PON1 activity in the serum may help to identify boars with reduced sperm quality, whereas the PON1 activity in the sperm-rich portion of the ejaculate may help to screen boars with high potential fertility. Future studies, with a greater number of animals and from different genetic lines should be conducted to validate PON1 activity as a fertility marker.
